# Comparative Genomics and Adaptive Evolution of *Bifidobacterium adolescentis* in Geographically Distinct Human Gut Populations

**DOI:** 10.3390/foods14152747

**Published:** 2025-08-06

**Authors:** Pei Fu, Hao Qi, Wenjun Liu

**Affiliations:** 1 Key Laboratory of Dairy Biotechnology and Engineering, Ministry of Education, Inner Mongolia Agricultural University, Hohhot 010018, China; fupei_12@163.com (P.F.); 15598011433@163.com (H.Q.); 2 Key Laboratory of Dairy Products Processing, Ministry of Agriculture and Rural Affairs, Inner Mongolia Agricultural University, Hohhot 010018, China; 3 Inner Mongolia Key Laboratory of Dairy Biotechnology and Engineering, Inner Mongolia Agricultural University, Hohhot 010018, China; 4 Collaborative Innovative Center of Ministry of Education for Lactic Acid Bacteria and Fermented Dairy Products, Inner Mongolia Agricultural University, Hohhot 010018, China

**Keywords:** *Bifidobacterium adolescentis*, comparative genomics, functional gene annotation, adaptive evolution

## Abstract

*Bifidobacterium adolescentis* is prevalent in the gastrointestinal tract of healthy humans, and significantly influences host health. Recent studies have predominantly investigated the probiotic characteristics of individual strains and their specific metabolic roles, whereas analyses at the population genome level have been limited to date. This study conducted a comparative genomics analysis of 543 *B. adolescentis* genomes to explore genetic background variations and functional gene differences across geographically diverse populations. The results revealed significant differences in genome size and GC content among populations from Asia, Europe, and North America (*p* < 0.05). The pan-gene exhibited an open structure, reflecting the substantial genetic diversity within *B. adolescentis*. Functional annotation demonstrated that *B. adolescentis* possesses numerous protein-coding genes and abundant carbohydrate-active enzymes (CAZys) implicated in carbohydrate degradation and transformation. Population-specific CAZys were identified, suggesting adaptive evolution driven by distinct regional dietary patterns.

## 1. Introduction

Bifidobacterium, a core constituent of the human intestinal microbiota, has consistently been a central focus of microbiological research since its initial discovery [1,2]. Recognized as one of the earliest dominant bacteria to colonize the human gastrointestinal tract, Bifidobacterium plays an irreplaceable role in various crucial physiological processes, including maintaining flora equilibrium, facilitating normal intestinal development, and safeguarding overall human health [3,4]. Among the genus Bifidobacterium, *Bifidobacterium adolescentis* is particularly prominent, and is consistently identified as the predominant species within the gastrointestinal tract of healthy individuals across various age groups [5]. *B. adolescentis* exhibits multiple probiotic characteristics, including anti-inflammatory effects [6], and the ability to metabolize diverse prebiotics, such as xylooligosaccharides [7], galactooligosaccharides (GOSs) [8] and arabinoglycans [9]. Furthermore, recent findings suggest that *B. adolescentis* has significant therapeutic potential in individuals with sarcopenia, enhancing muscle mass and function via niacin (NA) biosynthesis [10].

Comparative genomic analysis has emerged as a powerful tool for elucidating the genetic diversity, metabolic capabilities, and evolutionary relationships among microbial populations [11]. Applying comparative genomics to intestinal symbiotic bacteria, such as Bifidobacterium, facilitates comprehensive insights into strain-specific features and adaptations [12,13]. For example, the phylogenetic relationships between bacterial strains and their original environments can be accurately delineated based on genomic data, providing a deeper understanding of their ecological roles and adaptive strategies [14]. Additionally, comparative genomics combined with functional annotation significantly aids in elucidating the mechanisms underlying bacterial adaptation to distinct environmental niches [15]. Although significant progress has been made in understanding *B. adolescentis*, notable variations remain in terms of physiological functions, metabolic capacities, and effects on host health in different strains [16]. However, owing to limited genomic analyses, the underlying genetic determinants responsible for these differences have not been fully characterized. Consequently, genome sequencing and comprehensive functional genomic analyses of *B. adolescentis* are crucial for elucidating its genetic diversity, population structure, and evolutionary history, thereby enhancing its applicability in therapeutic and nutritional contexts.

In this study, we conducted genomic resequencing of 28 strains of *B. adolescentis* isolated and identified from fecal samples of adults collected in China. These newly generated genomic data, combined with 515 intestinal-derived *B. adolescentis* genomes available in the NCBI database (https://www.ncbi.nlm.nih.gov/), were systematically analyzed using bioinformatics methods. This comprehensive analysis encompassed genomic characterization, nuclear pan-gene determination, phylogenetic assessment, functional gene identification, and bacteriocin-encoding loci investigation. Collectively, our findings provide essential insights into the genetic diversity, adaptive evolutionary processes, and ecological specialization of *B. adolescentis*.

## 2. Materials and Methods

### 2.1. Strain Activation Culture

Activate 28 strains of *B. adolescentis* of human intestinal origin that were previously isolated and identified by our research team. Thaw the bacterial liquid of *B. adolescentis* mixed with skimmed milk in a sterile bench (Suzhou Sujie Chemical Equipment Co., LTD., Suzhou, China). Dip the bacterial solution and streak on the BHI AGAR medium (Qingdao High-tech Industrial Park Haibo Biotechnology Co., LTD., Hohhot, China). Incubate anaerobically at 37 °C for 48 h. (Single colonies were picked and cultured in BHI liquid medium for two generations, and then expanded for further culture.) Centrifuge the expanded culture bacterial liquid at a speed of 3800 r/min for 5 min and discard the supernatant. Wash the bacterial sludge twice with 20 mL of sterile PBS buffer solution, and then add 1.5 mL of PBS buffer solution. Shake and mix evenly. Aspirate the bacterial liquid into a 2 mL centrifuge tube. Centrifuge at 12,000 r/min for 2 min to collect the bacterial sludge. All reagents used in this experiment are analytical grade reagents (Shanghai Sinopharm Chemical Reagent Co., LTD., Hohhot, China).

### 2.2. Genome Sequencing and Assembly of Strains

The collected bacterial sludge was sent to Beijing Novozymes Biological Company for whole-genome sequencing. Sequencing was performed on the Illumina Novaseq 6000 high-throughput sequencing platform (Illumina, Inc., San Diego, CA, USA). Clean Data was obtained after removing low-quality data and connector sequences. Using the software of SOAPdenovo (V2.04), the appropriate Kmer value was selected to perform splicing assembly and single-base correction on the filtered data [17]. The assembly results were evaluated using genome size, GC content, Saffold quantity, N50 length and N90 length, as indicators. Finally, the sequences with better assembly results were selected for subsequent SOAP (V2.21) verification.

In addition, 515 genomic sequences of human intestinal-derived *B. adolescentis* were retrieved from the NCBI RefSeq (https://www.ncbi.nlm.nih.gov/datasets/genome/, accessed on 8 April 2025). Genome quality assessment was performed using CheckM (V1.1.9) [18], and genomes with genomic integrity below 95% and contamination levels exceeding 5% were excluded from further analyses (Table S1).

### 2.3. The Average Nucleotide Identity (ANI) Value Analysis

Average nucleotide identity (ANI) values were calculated for all 543 *B. adolescentis* strains, according to the methodology outlined by Goris [19].

### 2.4. Pan-Genome and Core-Genome Analysis and Phylogenetic Analyses

Genomic annotation was performed using the Prokka (V1.14.6) [20]. Pan-genome and core-genome analyses of the 543 *B. adolescentis* strains were constructed and visualized using PanGP (V1.0.1) [21]. A Neighbor-Joining (NJ) phylogenetic tree was constructed based on core-gene sequences using Treebest (V1.9.2) software with bootstrap analysis set at 1000 replicates.

### 2.5. Carbohydrate Activity Enzyme (CAZy) Analysis

Gene sequences from all strains were annotated for carbohydrate-active enzymes (CAZy). This annotation was integrated with data from the CAZy database (CAZy; http://www.cazy.org/, accessed on 30 April 2025) for further analyses.

### 2.6. Cluster of Orthologous Group (COG) Annotation

Protein sequence files (faa) generated by Prokka software annotation were compared with the Cluster of Orthologous Groups (COG; https://ecoliwiki.org/colipedia/index.php/Clusters_of_Orthologous_Groups_(COGs, accessed on 5 May 2025) database for functional categorization.

### 2.7. Prediction of Bacteriocin Manipulators

The potential bacteriocin-producing capabilities of the 543 *B. adolescentis* strains were predicted using the BAGEL4 website (http://bagel4.molgenrug.nl/) [22].

### 2.8. AntiSMASH Predictive Analysis

Gene clusters involved in secondary metabolite biosynthesis in *B. adolescentis* were annotated and analyzed using the antiSMASH website (https://antismash.secondarymetabolites.org/).

### 2.9. CRISPR Identification

The CRISPR sites in the genome of *B. adolescentis* were discovered using CRISPRCasTyper (V1.6.1) [23]. The type (Type I–VI) and subtypes (such as I-A, II-A, etc.) of the CRISPR-CAS system were identified. According to the nucleotide sequence of CRISPR repeat sequence, phylogenetic analysis was performed. The phylogenetic tree was constructed based on core-gene sequences, using Treebest (V1.9.2) software, with bootstrap analysis set at 1000 replicates.

### 2.10. Data Visualization and Statistics

Heat maps were generated using R (V4.2.2) software. Phylogenetic trees were visualized using the Interactive Tree of Life (iTOL; https://itol.embl.de/, accessed on 8 April 2025). Box plots, bar charts, and Venn diagrams were created using Origin 2024 software (V10.1.0.178). Statistical comparisons between datasets were performed using the Wilcoxon test. Statistical significance was set at *p* < 0.05.

## 3. Results

### 3.1. Genomic Features of B. adolescentis Isolates

Genome resequencing and assembly were conducted for 28 previously isolated *B. adolescentis* strains (Table S2). The genome size of 543 *B. adolescentis* strains ranged from 1.94 to 2.48 Mb, with an average size of 2.14 ± 0.09 Mb. The GC content varied between 58.58% and 60.29%, with an average GC content of 59.41 ± 0.28%. The predicted number of coding sequences (CDSs) ranged from 1626 to 2217, with an average size of 1833 ± 118. For strains sequenced by our research team, the average genome size was 2.10 ± 0.10 Mb, average GC content was 59.33 ± 0.20%, and average CDS count was 1797 ± 131. In contrast, the genome sequences retrieved from NCBI had an average genome size of 2.14 ± 0.09 Mb, an average GC content of 59.91 ± 0.29%, and an average CDS count of 1835 ± 117. The comparisons demonstrated that the genomic characteristics of the strains sequenced in this study closely aligned with those available in the NCBI database.

**Figure 1:**
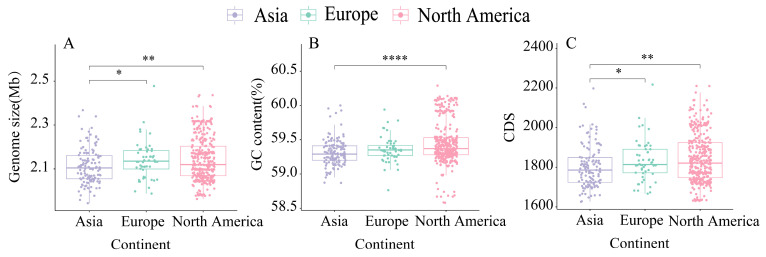
Genome comparison of 543 *B. adolescentis* strains across different continents. (**A**) Genome size; (**B**) GC content; (**C**) CDS. There is a statistically significant difference between the three groups represented by * (Wilcoxon test, significance level: * represents *p* < 0.05, ** represents *p* < 0.01, **** represents *p* < 0.0001).

Excluding the strain *B. adolescentis* ATCC 15703^T^, whose initial isolation site was unspecified, the remaining 542 *B. adolescentis* strains originated from 11 countries, including the United States, China, the United Kingdom, Italy, and Japan. These isolates comprised 125 strains from Asia, 52 from Europe, and 365 from North America. Notably, the genomic size and CDS counts of the Asian isolates were significantly lower than those of the European (*p* < 0.05) and North American (*p* < 0.01) isolates. Furthermore, the GC content of the Asian isolates was significantly lower than that of the North American isolates (*p* < 0.001). Collectively, these results confirmed the regional differences in genome size, GC content, and CDS counts among *B. adolescentis* strains isolated from diverse geographical populations (Figure 1).

### 3.2. ANI Values of B. adolescentis

**Figure 2:**
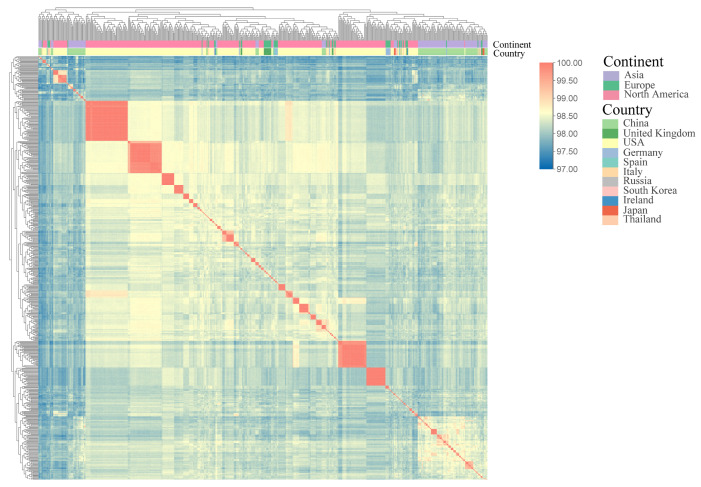
ANI heat map of *B. adolescentis*, using the color coding of the genome on the x-axis to distinguish where strains were isolated.

ANI is a robust indicator for assessing genomic correlations among bacterial species, with a generally accepted species-delineation threshold between 95 and 96% [24]. In the present study, the ANI values among all *B. adolescentis* isolates exceeded 97%, and the ANI values relative to the type strain *B. adolescentis* ATCC 15703^T^ averaged 97.87% ± 0.12%. Thus, all isolates analyzed in this study belonged to *B. adolescentis*. A clustering heatmap of the isolates revealed evident geographical clustering patterns, indicating regional genomic variations among isolates from different geographical locations (Figure 2).

### 3.3. Pan–Core Genome of B. adolescentis

**Figure 3:**
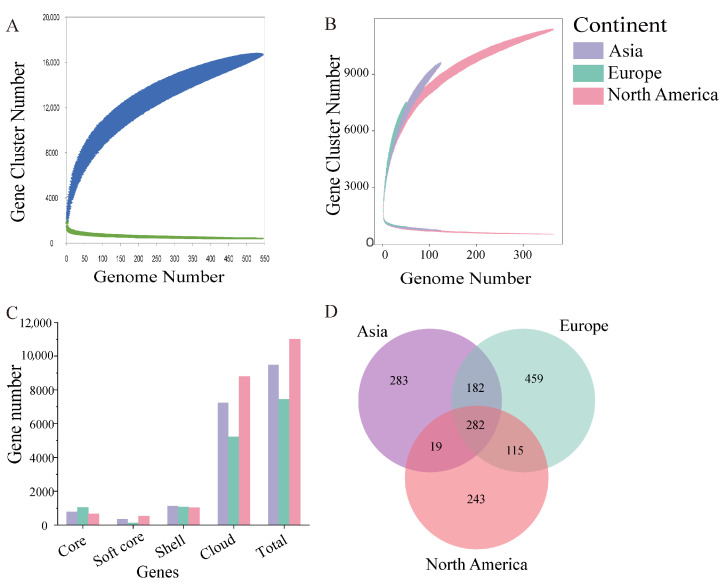
*B. adolescentis* pan–core gene. (**A**) Overall pan–core gene trend map; (**B**) pan–core gene trend map of the three continents; (**C**) pan–core gene comparison of isolates from different regions; (**D**) Venn map of core genes of isolates from different regions.

The pan-genome of 543 *B. adolescentis* strains comprised 16,696 genes, including 412 core genes (2.47%), 4662 unique genes (27.92%), and 11,622 ancillary genes (69.61%). The core-genome curve demonstrated an initial rapid decline, followed by gradual stabilization as the number of genomes analyzed increased, whereas the pan-genome curve remained open, indicating ongoing genomic expansion (Figure 3A). Notably, the pan-gene curves varied among strains originating from different geographical locations, with Asian isolates exhibiting a significantly higher pan-genome expansion than North American isolates (Figure 3B).

A comparative analysis of pan–core genomes among geographically distinct *B. adolescentis* populations revealed that North American isolates exhibited the largest dataset, characterized by the highest number of pan genes and the fewest core genes. Conversely, European isolates comprised the smallest dataset, with the fewest pan genes and the highest number of core genes (Figure 3C). Venn diagrams depicting the core-gene sets among geographic isolates identified 282 common genes across all regions (Figure 3D). European isolates, despite being the smallest in number, had the greatest number of unique core genes, exhibiting the most pronounced divergence compared with North American isolates.

### 3.4. The Phylogenetic Tree of B. adolescentis

**Figure 4:**
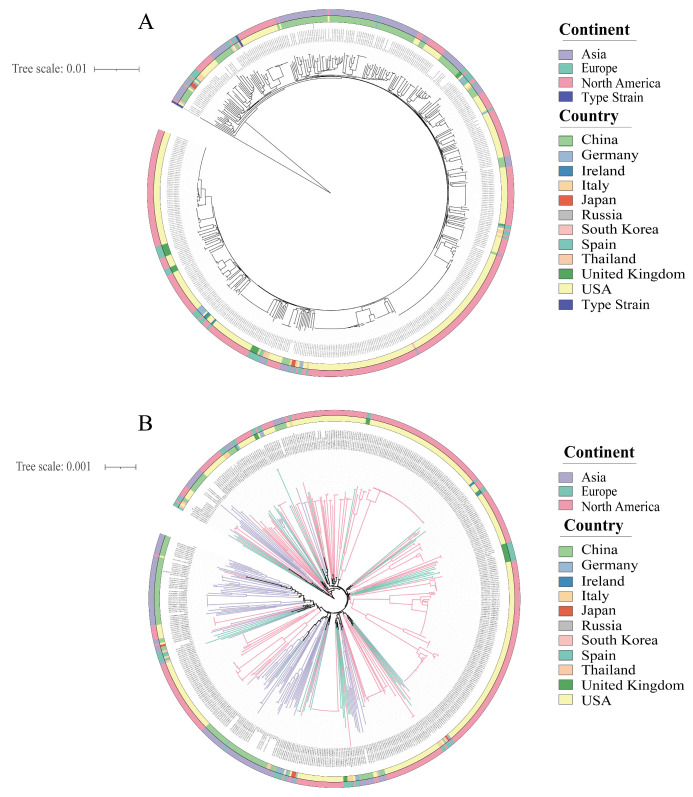
Phylogenetic tree: (**A**) with outgroup phylogenetic tree and (**B**) without outgroup phylogenetic tree.

The phylogenetic relationships among *B. adolescentis* strains were assessed using *Bifidobacterium ruminantium* LMG 21811^T^ [25], which is closely related to *B. adolescentis*, as an outgroup (Figure 4A). Branch lengths reflected genetic distances, clearly distinguishing the 543 *B. adolescentis* strains from the outgroup and confirming their classification as a single species. Additionally, a radial phylogenetic tree constructed based on 413 core genes from 542 *B. adolescentis* strains (excluding the outgroup) showed distinct clustering patterns (Figure 4B). Asian and North American isolates predominantly formed distinct clusters, whereas European isolates appeared more dispersed, but displayed partial clustering tendencies. At the national scale, the strains generally clustered according to their country of origin. Within Europe, isolates from Italy, Spain, and the United Kingdom formed a coherent cluster, consistent with the clustering observed, based on the ANI values.

### 3.5. Annotation of Carbohydrate Enzyme in B. adolescentis

**Figure 5:**
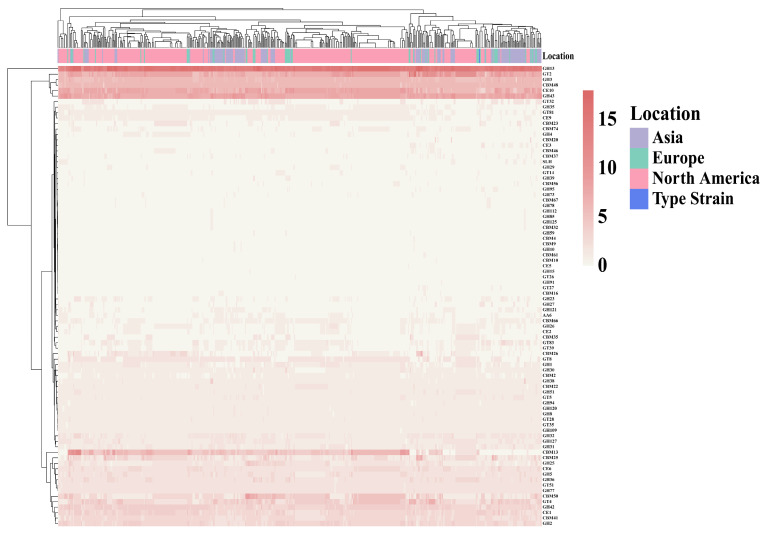
Carbohydrate analysis of 543 strains of *B. adolescentis*.

To elucidate the carbohydrate utilization pathways in *B. adolescentis*, we annotated CAZy from 543 genomes based on the CAZy database. The annotation revealed a diverse array of CAZy present within the *B. adolescentis* genome (Figure 5). A total of 90 distinct CAZy families were identified, comprising 59 glycoside hydrolases (GHs), 14 glycosyltransferases (GTs), 13 carbohydrate-binding modules (CBMs), and 4 carbohydrate esterases (CEs), The results are shown in Table S3. The GH family predominated, constituting 65.56% of the total CAZymes. Within the GH family, GH3, GH42, and GH2 were the most abundant, significantly exceeding other GH family members. These enzymes predominantly participate in carbohydrate metabolism and β-glycosidic bond hydrolysis, thereby aiding the host in the digestion of dietary fibers and lactose. Among the GTs, GT2_Glycos_transf_2 exhibited significantly higher abundance than other GT family members, playing a critical role in synthesizing polysaccharides, such as peptidoglycan, glycogen and exopolysaccharides. Regarding CBMs, the numbers of CBM13, CBM26 and CBM48 are significantly higher than those of other members. All of them can enhance the affinity of enzymes for substrates, and improve catalytic efficiency. Within the CE family, CE10 is predominant, and primarily facilitates the degradation of cellulose and hemicellulose.

Comparative analysis of CAZy gene distribution revealed the greatest diversity in North American isolates, followed by Asian and European isolates. Furthermore, 15 CAZy genes were geographically specific: 11 GHs, 3 CBMs, and 1 GT. Specifically, Asian isolates possessed CBM32, CBM50, GH112, GH125, GH5_18, and GH85; European isolates featured GH30_9 and GH59; whereas North American isolates harbored CBM34, GH146, GH15, GH39, GH43_4, GH91, and GT26.

### 3.6. Annotation of COG in B. adolescentis

**Figure 6:**
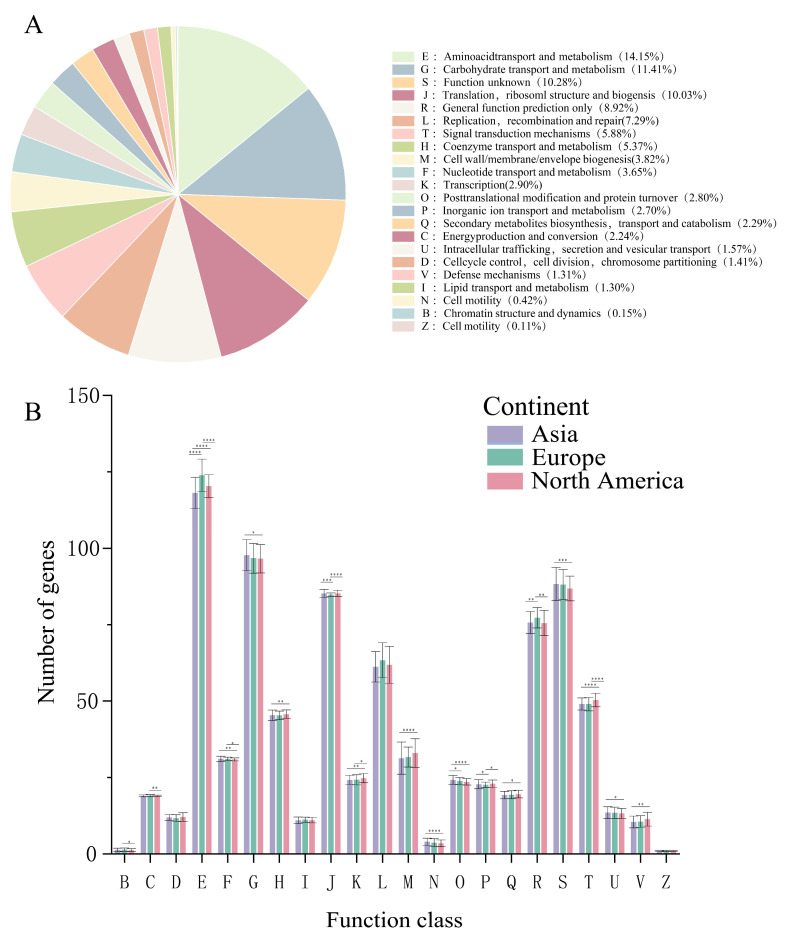
COG annotation results of *B. adolescentis*. (**A**) COG annotation results of 543 *B. adolescentis* strains; (**B**) COG annotation results for strains from different isolation sites. There is a statistically significant difference between the three groups represented by * (Wilcoxon test, significance level: * represents *p* < 0.05, ** represents *p* < 0.01, *** represents *p* < 0.001, **** represents *p* < 0.0001).

Functional annotation of Clusters of Orthologous Group (COG) genes was conducted for 543 *B. adolescentis* strains (Figure 6A). Annotation identified four major functional categories, encompassing 22 functional classifications (Table S4). Genes associated with metabolic processes constituted the largest proportion, accounting for 43.11% of the annotated genes. Among the metabolic genes, family E was the most prevalent (14.15%), followed by family G (11.41%). Genes related to information storage and processing comprised 20.37% of the total, with family J representing the highest proportion (10.03%). Additionally, genes involved in cellular growth and signal transduction accounted for 17.33% of the genes, predominantly represented by the family T (5.88%). Genes with unclassified or unknown functions comprised 19.20% of the total, with families S and R contributing 10.28% and 8.92%, respectively. Thus, aside from genes of unknown function, the predominant functional categories in *B. adolescentis* included amino acid metabolism and transport, carbohydrate metabolism and transport, translation, ribosome structure and biogenesis, and DNA replication, recombination, and repair.

Analysis of isolates from different geographical regions indicated significant variations in the abundance of families E, G, J, L, T, H, R, and S. Families S and R, which lack clearly defined functions, were excluded from subsequent comparative analyses because of ambiguity regarding their specific roles (Figure 6B).

**Figure 7:**
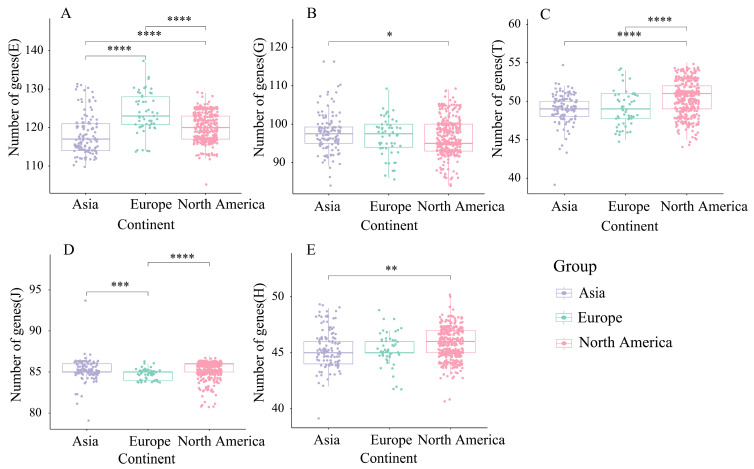
COG functional annotation of different isolated strains (**A**–**E**): analysis of significant differences in genes with different functions. Wilcoxon test, significance level: * for *p* < 0.05, ** for *p* < 0.01, *** for *p* < 0.001, **** for *p* < 0.0001.

Consequently, differences among families E, G, J, L, T, and H were further analyzed (Figure 7). For family E, European isolates showed a significantly higher abundance than Asian and North American isolates (*p* < 0.0001). In family G, the number of Asian isolates significantly exceeded that of North American isolates (*p* < 0.05). Family T genes were significantly more abundant in North American isolates than in Asian and European isolates (*p* < 0.0001). For family J, isolates from Asia differed significantly from European isolates (*p* < 0.001), and North American isolates differed more significantly from European isolates (*p* < 0.0001). Finally, family H genes were significantly more abundant in North American than in Asian isolates (*p* < 0.01).

### 3.7. Annotation of Bacteriocin Operon in B. adolescentis

**Figure 8:**
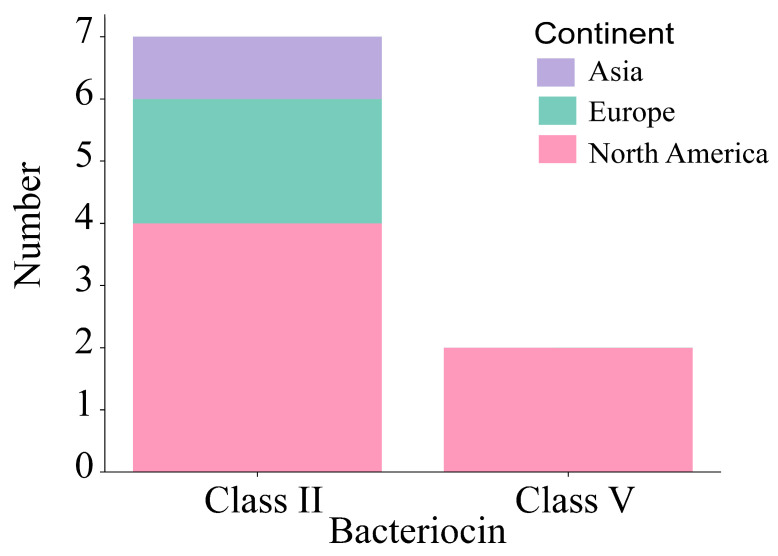
Types of bacteriocin operon.

Among the 543 *B. adolescentis* strains analyzed, nine bacteriocin-related operons were annotated. Of these, seven operons encoded Class IIa bacteriocins, including Propionicin_SM1, whereas two operons encoded Class V bacteriocins, such as pesticin. The bacteriocin manipulators exhibited geographical variation, annotated in one Asian strain, two European strains, and six North American strains (Figure 8). Notably, Class V bacteriocin manipulators were exclusively identified among North American isolates, further suggesting regional differences in genomic diversity.

### 3.8. AntiSMASH Predictive Analysis by B. adolescentis

In addition to bacteriocins, which are antimicrobial peptides secreted by bacteria as part of their defense mechanisms, microorganisms also employ non-peptide secondary metabolites, such as terpenoids, polyketides, and nonribosomal peptides (NRPs), for competitive niche occupation [26]. Annotation of gene clusters related to secondary metabolite synthesis in *B. adolescentis* strains identified 556 gene clusters. Each strain possessed gene clusters associated with terpene precursor metabolism. Terpene precursors are crucial intermediates in terpenoid biosynthesis, underpinning various microbial secondary metabolites and influencing human health through mechanisms such as protein modification, immune regulation and microbiota interactions.

**Figure 9:**
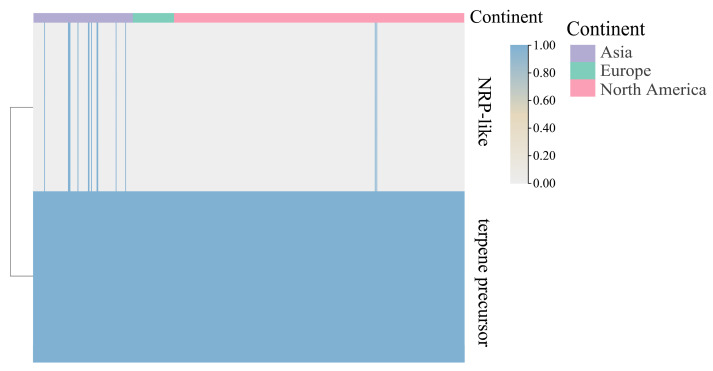
Heatmap of the distribution of gene clusters related to the synthesis of other secondary metabolites.

Additionally, gene clusters associated with NRP-like metabolism, which is integral to the biosynthesis and modification of small-molecule metabolites [27], were also annotated. NRP-like pathways are important for synthesizing medically and industrially relevant compounds, including antibiotics, antifungal agents and immunosuppressants. Remarkably, these NRP-like clusters exhibited distinct geographical clustering: among the 14 identified clusters, 12 were exclusive to Asian isolates, 2 were present in North American isolates, and European isolates lacked NRP-like gene clusters entirely (Figure 9).

### 3.9. Identification of CRISPR-Cas Systemn in B. adolescentis

**Figure 10:**
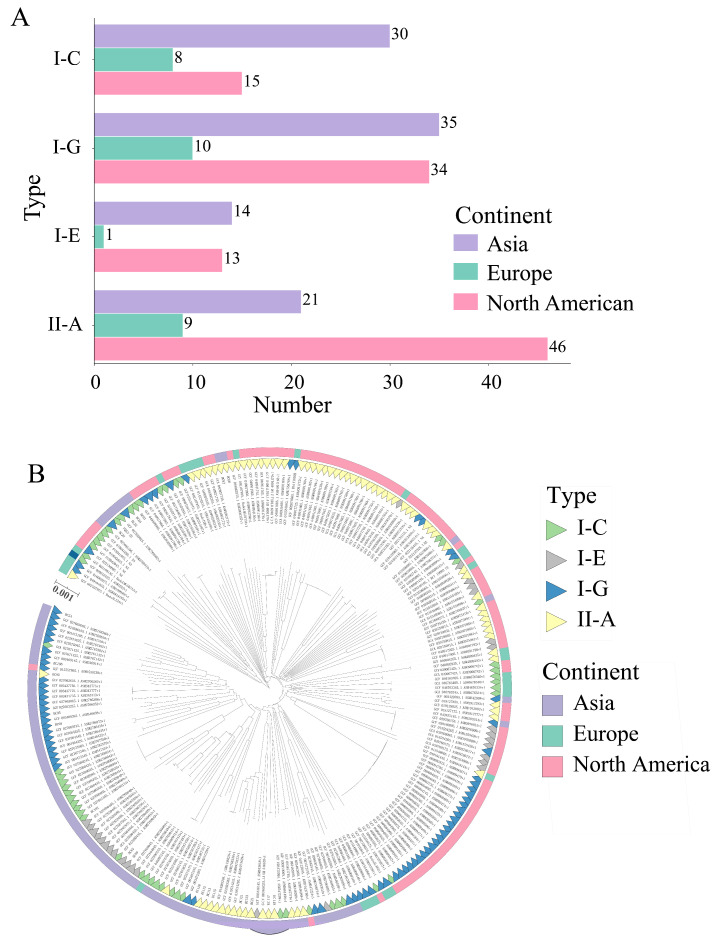
Identification using CRISPR-Cas system in *B. adolescentis*. (**A**) CRISPR-Cas system identification results; (**B**) phylogenetic analysis and subtype distribution of *B. adolescentis*.

The immune defense characteristics of *B. adolescentis* were analyzed by CRISPR-Cas. The research found that, among 543 strains of *B. adolescentis*, the genomes of 237 strains encoded the CRISPR-Cas system based on the presence of Cas marker proteins, including 100 Asian isolates, 108 North American isolates and 28 European isolates. There are four subtypes of CRISPR-Cas, namely I-G (78 strains), I-C (59 strains), I-E (27 strains), and II-A (73 strains) (Table S5). Type I systems (30.20%) have more categories than Type II systems (13.44%). Isolates from Asia, Europe and North America all have the above subtypes. The I-G content was the highest in the isolates from Asia and Europe, and the II-A content was the highest in North America (Figure 10A). Phylogenetic trees were constructed for strains identified by the CRISPR-Cas system (Figure 10B). It was found that under the same branch, most strains were encoded as homologous systems. This indicates that the repetitive sequences of the same type of CRISPR-Cas system have a high degree of homology on the phylogenetic tree.

## 4. Discussion

Previous studies on *B. adolescentis* have predominantly emphasized specific functional aspects of individual strains, such as intestinal colonization, immune regulation, metabolic regulation, and probiotic strain development, with limited exploration of population genomics. In this study, we analyzed 28 strains of *B. adolescentis* isolated from the human gut, integrating these findings with data from 515 high-quality genomic sequences of *B. adolescentis* sourced from the NCBI database, which were also isolated from the human gut. The geographic distribution of these isolates included 125 strains from Asia, 52 from Europe, and 365 from North America. Comparative genomic analysis was employed to elucidate evolutionary trajectories, providing a basis for subsequent genomic and functional gene studies related to *B. adolescentis*.

In comparative genomics, ANI values are commonly used to assess genomic polymorphisms and similarities among bacterial species [28]. Our ANI analysis of 543 *B. adolescentis* strains confirmed species identity, demonstrating values consistently exceeding 97%, which is indicative of high genome similarity with a tendency toward regional clustering. Notably, Asian isolates exhibited significantly smaller genome sizes and fewer CDS than European and North American isolates. Furthermore, the GC content in Asian isolates was significantly lower than that observed in North American isolates, underscoring the distinct regional genomic variation within *B. adolescentis* populations. A similar pattern of regional genomic divergence was documented by Wu et al. in their comparative genomic assessment of 247 *B. pseudocatenulatu* strains [29], wherein Chinese isolates demonstrated smaller genomes relative to Japanese and Vietnamese isolates (*p* < 0.05), with Vietnamese isolates exhibiting the highest CDS numbers. ANI values within populations from the same country significantly surpassed those observed between countries, mirroring our findings and reinforcing the notion of geographically driven genomic evolution.

Owing to substantial intraspecific genomic heterogeneity, bacterial pan-genomes typically remain open, indicating frequent genetic exchange between species [30]. Conversely, closed pan-genomes, which are characterized by restricted gene flow, are rarely observed [31]. Our findings indicate that *B. adolescentis* possesses an open pan-genome, highlighting its considerable environmental adaptability. A total of 282 core genes were identified across various geographical isolates, with European isolates harboring the largest and North American isolates the smallest core-gene pool. Core genes are critical for fundamental biological functions, and determine the essential phenotypic characteristics and underlying adaptive traits of the microbial communities [32,33]. Collectively, our results suggest that *B. adolescentis* has undergone adaptive evolutionary processes in geographically distinct human gut environments, resulting in significant genetic diversity. This adaptive genomic evolution, driven by local ecological niches, underpins the genomic variability observed among regional *B. adolescentis* populations. Phylogenetic analyses based on core genes further reinforced these observations, delineating clear correlations between phylogenetic relationships and geographic segregation.

Carbohydrates are the primary energy source that supports the growth and metabolic functions of bacterial strains. CAZys are a diverse family of enzymes capable of specifically recognizing, degrading, and synthesizing carbohydrates, and play pivotal roles in human health, nutrition, intestinal microbiota dynamics, and plant–pathogen interactions [34]. Among the 543 *B. adolescentis* strains examined, GHs were the most frequently annotated enzymes, followed by GTs. This finding aligns closely with the results of Sun et al., who annotated CAZymes in 213 lactic-acid bacteria strains and reported that GHs and GTs were the predominant enzyme classes [35]. The genetic diversity of CAZys was highest in North American isolates, followed by Asian strains, and lowest in European ones. Each geographical region harbored a specific CAZy profile. Specifically, Asian isolates contained distinct enzymes, such as CBM32, CBM50, GH112, GH125, GH5_18, and GH85, associated with the degradation of complex substrates, including galactoglycan [36], glycoprotein, and cellulose [29]. These enzymes facilitate the digestion of soy and dairy products prevalent in Asian diets. European isolates uniquely annotated GH30_9 and GH59, which are enzymes beneficial for dietary fiber fermentation and cereal digestion, reflecting a dietary preference in Europe, where bread is a dietary staple. North American isolates specifically annotated CBM34, GH146, GH15, GH39, GH43_4, GH91, and GT26, promoting fiber degradation, enhancing sugar metabolism, and aiding the digestion of high-sugar foods, which is consistent with the dietary patterns typical in North America. These findings underscore the adaptive evolution of *B. adolescentis*, which is similar to the utilization of oligosaccharides (HMOs) in breast milk, by infants. When breastfeeding an infant, the host’s diet consists entirely of (HMO-rich) breast milk, which is an indigestible carbohydrate [37]. The adaptation to metabolic HMOs promotes the dominance of specific bifidobacterium groups in the intestinal microbiota of newborns. Weaning drives a gradual succession of bifidobacterial taxa from those tuned for HMO utilization to those more adapted to foraging dietary glycans (oligo- and polysaccharides) of plant origin [38]. This is caused by dietary differences.

To further understand the main functions encoded by the genes in the 543 *B. adolescentis* strains, COG functional annotation was performed. *B. adolescentis* exhibited a rich repertoire of protein-coding genes, predominantly associated with metabolic processes, followed by genes implicated in information storage and modification. Among the geographical variants, notable differences were observed primarily in families E, G, and J. Specifically, the G family genes linked to carbohydrate metabolism and transport were highly prevalent in Asian isolates, reflecting their adaptation to the carbohydrate-rich Asian diet. Conversely, European isolates exhibited significantly elevated E family gene content, primarily involved in amino acid metabolism and transport, which aligns with a diet rich in protein sources, such as grains, fish, and cheese. These differences likely stem from the distinct dietary environments that shaped the nutritional conditions encountered by the strains, further highlighting the adaptive and evolutionary processes in *B. adolescentis*.

Bacteriocins are ribosomally synthesized proteinaceous or polypeptide metabolites that exhibit antibacterial activity, enhancing bacterial competitiveness within the gastrointestinal tract by suppressing bacterial growth [39,40]. Among the annotated 543 *B. adolescentis* strains, 7 Class IIa bacteriocins and 2 Class V bacteriocins were identified. As pivotal intestinal probiotics, Bifidobacterium species primarily maintain gut microbiota equilibrium [41] by inhabiting the intestinal niche through long-term symbiotic relationships with the host and other microorganisms. The survival strategy of Bifidobacterium predominantly emphasizes adherence and colonization capabilities [42], rather than bacteriocin-mediated antagonism. Consequently, the relatively limited annotation of only nine bacteriophage manipulators in 543 *B. adolescentis* strains supports this hypothesis.

In prokaryotes, the CRISPR gene and its associated Cas protein together form an adaptive immune system used to defend against the invasion of exogenous genetic material (such as bacteriophages or plasmids) [43]. Many strains with probiotic potential may be restricted in terms of colonization ability and other aspects, during the process of development and application. The gene-editing technology of CRISPR-Cas can break some genetic limitations, thereby altering certain biological characteristics of strains, and enhancing their application value [44]. At present, there are few studies on CRISPR of Bifidobacterium. Among all the CRISPR types studied, Cas1 and Cas2 are essential for obtaining spacer sequences [45]. Cas3 and Cas9 are, respectively, the characteristic genes of type I and type II, used to distinguish different subtypes. This study predicted the CRISPR-Cas system of 543 strains *B. adolescentis*. It was found that 43.65% (237/543) of the genome encoded the complete CRISPR-Cas system. A total of four subtypes were identified, and the above CRISPR-Cas systems were present in isolates from Asia, Europe and North America. The CRISPR-Cas systems of isolates from different regions have different quantitative advantages. Among the isolates from North America, type II-A has the highest content. Among the isolates from Asia and Europe, the I-G type has the highest content. Therefore, to a certain extent, the CRISPR-Cas system has geographical relevance. However, it is still necessary to further explore the formation mechanism and evolutionary driving factors behind it. Future research can combine a broader range of geographical samples, metagenomic analysis, and evolutionary biology methods, to deeply understand the geographical distribution patterns and adaptive significance of the CRISPR-Cas system.

## 5. Conclusions

This study examined the genomic features of 543 *B. adolescentis* strains from geographically distinct populations, using comparative genomic analysis and functional gene annotation. Significant differences were identified in the core-gene compositions, with phylogenetic analyses based on these genes revealing clear geographical clustering, indicative of genetic diversity. The annotation of strains with CAZys and COG revealed distinct regional enzymatic profiles. Specifically, Asian isolates possessed six specific enzymes, including CBM32 and CBM50, which are associated with glycoproteins and cellulose degradation, facilitating the digestion of soy and dairy products. Moreover, Asian isolates exhibited a higher abundance of G-family proteins, predominantly involved in carbohydrate metabolism and transport, likely reflecting a high-carbohydrate regional diet. European isolates contain specific enzymes, GH30_9 and GH59, which enhance dietary fiber fermentation, are beneficial for cereal digestion, and exhibit elevated E-family protein levels, potentially correlated with a high-protein diet. North American isolates were annotated with seven specific enzymes, including GT26, associated with enhanced glucose metabolism, possibly reflecting the high-sugar diet of this region. The correlation between annotated functional genes and dietary structures supports the hypothesis that *B. adolescentis* has undergone adaptive evolution in response to localized geographical dietary differences. These findings enhance our genetic understanding of *B. adolescentis* and provide foundational knowledge for future investigations of its genomic and functional characteristics.

## Data Availability

Raw sequence data are available in the NCBI sequence read archive (project PRJNA1281297). The data can be obtained from the NCBI database (https://www.ncbi.nlm.nih.gov/datasets/genome/?taxon=1680, accessed on 8 April 2025).

## Supplementary Materials

The following supporting information can be downloaded at: https://www.mdpi.com/article/10.3390/foods14152747/s1. Table S1. Basic information of 515 strains of *B. adolescentis* in NCBI; Table S2. Basic information of 28 strains of *B. adolescentis*; Table S3. CAZys annotation results of 543 strains of *B. adolescentis*; Table S4. COG annotation results of 543 strains of *B. adolescentis*; Table S5. Identification information of CRISPR-Cas Systemn for *B. adolescentis*.

## Abbreviations

The following abbreviations are used in this manuscript:

CAZys	carbohydrate-active enzymes
GOSs	galactooligosaccharides
NA	niacin
ANI	average nucleotide identity
NJ	Neighbor-Joining
COG	Cluster of Orthologous Groups
CDS	predicted number of coding sequences
GHs	glycoside hydrolases
GTs	glycosyltransferases
CBMs	carbohydrate-binding modules
CEs	carbohydrate esterases
NRPs	nonribosomal peptides
